# Regulatory effects of lncRNAs and miRNAs on autophagy in malignant tumorigenesis

**DOI:** 10.1042/BSR20180516

**Published:** 2018-10-23

**Authors:** Qingqing Yin, Wei Feng, Xianjuan Shen, Shaoqing Ju

**Affiliations:** 1 Center of Laboratory Medicine, Affiliated Hospital of Nantong University, Nantong, China; 2 Surgical Comprehensive Laboratory, Affiliated Hospital of Nantong University, Nantong, China

**Keywords:** autophagy, large intervening non-coding RNA, microRNA, oncogenesis, gene therapy

## Abstract

Autophagy is an important process in endogenous substrate degradation by lysosomes within cells, with a degree of evolutionary conservation. Like apoptosis and cell senescence, cell autophagy is a very important biological phenomenon involving the development and growth of biological processes. Abnormal autophagy may lead to tumorigenesis. In recent years, increasing studies have demonstrated that long non-coding RNAs (lncRNAs) and miRNAs can regulate cell autophagy by modulating targetting gene expression. In this review, we will provide an overview of lncRNAs and miRNAs in autophagy modulation and new insights into the underlying mechanisms, as well as their potential utilization in disease diagnosis, prognosis, and therapy.

## Introduction

**Figure 1:**
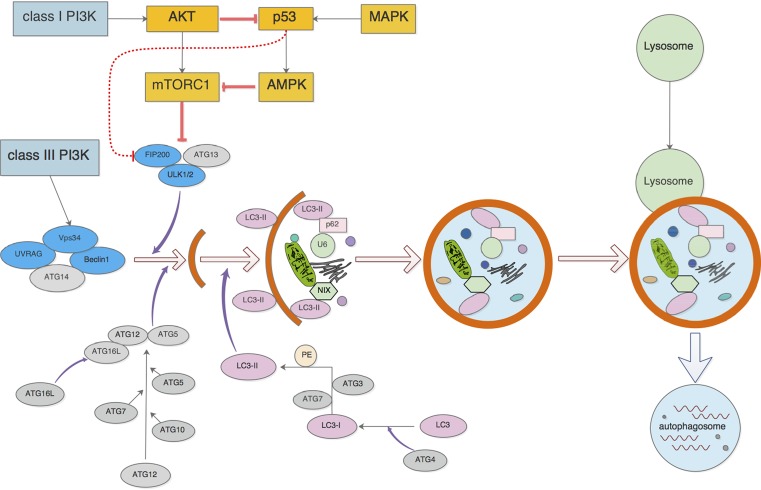
The process of autophagy and related regulatory proteins and signaling pathways Oncogenic and tumor suppressive signaling pathways are closely related to autophagy initiation. MAPK signaling can activate autophagy through AMPK activation and promotion of autophagy-related gene translation. PI3K-AKT signaling inhibits autophagy through mTOR activation and p53 inhibition. The Vps34 complex combines Beclin-1 promoting autophagic cell death which threatens the survival of cells. The linkage of ATG5 to ATG12 and ATG16L, and phosphatidyl ethanolamine (PE) to proteins of the microtubule-associated protein 1 light chain 3 (LC3) are vital for the initiation and development of autophagy.

Autophagy is a process of sustaining metabolism and homeostasis by capturing and degrading intracellular components such as proteins and organelles. Autophagy plays an important role in protein and organelle quality control, knowing that a low level of basal autophagy can prevent gradual accumulation of damaged proteins and organelles in tissues, which is known to be toxic over time [1]. Three forms of autophagy are commonly described: macro-autophagy, micro-autophagy, and chaperone-mediated autophagy (CMA). Autophagy is an important cellular response to stress or starvation. The formation and development of autophagy involve many signaling pathways and related proteins (Figure 1), and the regulation of these signaling pathways and proteins can affect the process of autophagy [2]. In the initiation step of autophagy, the widely accepted sensor is the mechanistic target of rapamycin (mTOR) complex I (mTORCI) and many autophagy inducers trigger autophagy by initiating signal transduction cascades to tactfully inhibit mTORCI [3]. mTORCI plays a central role in the regulation of ULK1-ATG13-FIP200 (*FAK family-interacting protein of 200 kDa*) complex where inhibition of mTORCI upon rapamycin treatment enhances the kinase activity of ULK1 [4]. Phosphoinositide 3-kinases (PI3Ks) are divided into three isoforms (class I, class II, and class III). The class I PI3K triggers mTOR signaling pathway and inhibits autophagy, while the class III isoform activates the autophagy by corresponding to Vps34. The contribution of class II PI3K activity on autophagy is unclear [5]. Autophagy has been reported to either inhibit or promote cancer cell proliferation or tumorigenesis in model systems, suggesting that the role of autophagy in cancer is context dependent [6,7]. Moderate and effective autophagy can remove tumor cells and maintain homeostasis. However, impaired autophagy may reduce cell viability, and delayed elimination of apoptotic cells from the body may induce the development of cancer [8].

Advances in human genome sequencing have shown that more than 90% of the human genomes are extensively transcribed but only approximately 2% of them serve as protein-coding genes [9]. The majority of the remaining transcripts known as non-coding RNAs (ncRNAs) have no protein-coding capacity, including small ncRNAs, especially miRNAs, and long ncRNAs (lncRNAs) based on their transcript size. LncRNAs are RNA transcripts that can be transcribed by polymerase II or III and are usually longer than 200 nts in length. Accumulating evidence shows that lncRNAs participate in the regulation of cell proliferation and apoptosis, and play crucial roles in tumorigenesis, progression, and drug resistance [10]. MiRNAs are endogenous RNAs, 19–25 nts in length, playing important gene-regulatory roles by pairing with the mRNAs of protein-coding genes to direct their post-transcriptional repression [11]. MiRNAs are reported to be dysregulated in the progression and invasion of various human cancers and play key roles in cell differentiation, proliferation, and cell death [12].

In recent years, increasing studies have demonstrated that lncRNAs and miRNAs can regulate cell autophagy *in vitro* and *in vivo* by modulating targetting gene expression at the level of chromatin organization, transcription, and post-transcription [13]. This review will provide an overview of lncRNAs and miRNAs in autophagy modulation and new insights into the underlying mechanisms of the lncRNA-autophagy axis and miRNA-autophagy axis and lncRNA-miRNA-autophagy axis in tumorigenesis.

## Roles of miRNAs in regulating autophagy

The effect of miRNA on target gene mRNA depends mainly on the degree of complementarity between miRNA and target gene transcriptional sequence, mainly through three ways. The first is to cut-off the mRNA molecule of the target gene, knowing that miRNA is completely complementary to the target gene, which is similar to the siRNA, and finally cut the target mRNA. The second is to inhibit translation of the target gene, which is not completely complementary to the target gene, and suppress the translation without affecting the stability of mRNA. The third is binding inhibition, which has the above two modes: when combined with the target gene, it directly and targettedly cuts mRNA; when the target gene is not fully integrated, it plays a role in regulating gene expression [14].

### miRNAs target autophagy-related proteins to regulate the autophagy

Beclin1 (ATG6) is a well-known key regulator of autophagy. Beclin1 can restore starvation-induced autophagy in ATG6-disrupted yeast strains and human breast carcinoma cells lacking detectable Beclin1 levels, whereas Beclin1 overexpression activates autophagy. Sufficient levels of Beclin1 are necessary for its autophagic function [15]. miR-30a, a member of the miR-30 family, is known to target Beclin1, and its expression was found to down-regulate Beclin1 expression and inhibit autophagy in medulloblastoma cell lines [16]. MiR-21 was up-regulated in chronic myeloid leukemia (CML), and treatment with antimiR-21 increased autophagy-related proteins Beclin-1, Vps34, and light chain 3 (LC3) II (LC3-II), eventually leading to an increase in autophagy flux [17].

Altered expression of miRNAs under hypoxia may induce a pathological process via modulating the expression of their downstream genes [18]. Hypoxia-induced up-regulation of miR-301a/b increased cell autophagy and viability of prostate cancer (PCa) cells by targetting *N-myc downstream regulated gene 2* (*NDRG2*) which could lead to the increasing LC3-II [19]. In addition, Yu et al. [20] demonstrated that miR-24-3p accelerated cell proliferation, migration, invasion, and autophagy and inhibited apoptosis by inhibiting *death effector domain containing* (*DEDD*) and activating p62 (a crucial autophagy factor, play irreplaceable roles in eliminating the ubiquitinated damaged mitochondria by mitophagy) in bladder cancer. *Myotubularin related protein 3* (*MTMR3*) is a member of myotubularin-related protein family, and has been demonstrated that *MTMR3* decreased *pattern recognition receptor* (PRR)-induced PI3P and autophagy levels by targetting ATG5 and LC3-II in monocyte-derived macrophages [21]. miR-181a was found to be significantly up-regulated, while *MTMR3* was found to be down-regulated in human gastric cancer (GC) tissues and cell lines [22]. Further investigations indicated that overexpression of miR-181a or depletion of *MTMR3* attenuated starvation-induced autophagy in adenogastric sarcoma (AGS) cells, resulting in an increase in cell proliferation, colony formation, migration, invasion, as well as suppression of apoptosis [23].

Sirtuin 1 (Sirt1) is a histone deacetylase and mediates autophagy via LC3. Further research demonstrated that Sirt1-induced autophagy could deacetylate endogenous LC3 [24]. Deacetylation of LC3 by Sirt1 allows LC3 to interact with the nuclear protein DOR and return to the cytoplasm with DOR, where it is able to bind ATG7 and other autophagy factors and undergo phosphatidyl ethanolamine conjugation to preautophagic membranes. The association of deacetylated LC3 with autophagic factors shifts LC3’s distribution from the nucleus toward the cytoplasm [25]. The mimics of miR-152 and miR-24 induced autophagy by increasing the level of Sirt1 and deacetylated LC3, which may be important in preventing the development of uterine sarcoma [26]. In A549 and H460 cells of lung cancer, by increasing the levels of both LC3-II and Beclin1, miR-144 targetted *the p53-induced glycolysis and apoptosis regulator* (*TIGAR*), inhibited proliferation, enhanced apoptosis, and increased autophagy [27].

Smad2, a key downstream component of the TGF-β signaling pathway, was found to contribute to cancer initiation, invasion, metastasis, and self-renewal of colorectal cancer (CRC) stem cells [28]. MiR-140-5p could directly target Smad2 and its overexpression was found to decrease the Smad2 expression level, thus attenuating cell invasion and proliferation. Ectopic expression of miR-140-5p in cancer stem cells (CSCs) inhibited CSC growth and sphere formation *in vitro* by disrupting autophagy through suppressing ATG12 [29]. Recently, autophagy has been suggested to inhibit HPV infection, which is known to be closely related to cervical cancer. Further analysis revealed that increased level of miR-224-3p expression inhibited autophagy through targetting FIP200 in cervical cancer [30].

### miRNAs target signaling pathways to regulate the autophagy

*X-linked inhibitor of apoptosis* (*XIAP*) was found to promote cell survival and suppress autophagy through XIAP-Mdm2-p53 pathway [31]. Forced expression of miR-23a significantly decreased the expression of *XIAP* and promoted autophagy, while down-regulation of miR-23a increased *XIAP* expression and suppressed autophagy in breast cancer cells [32]. But overexpressed miR-23a in melanoma suppressed the invasive and migratory properties of melanoma cells by abrogating autophagy through directly targetting ATG12 via autophagy-mediated AMPK-RhoA pathway [33].

As a protein kinase, mTOR is a confluence of upstream pathways regulating cell growth, proliferation, survival, and autophagy. It was reported [34] that activation of the mTOR pathway inhibited autophagy, indicating that some miRNAs can affect the process of cancers by regulating autophagy via the mTOR pathways. It was reported that the AMPK-mTOR signaling pathway regulated cell autophagy in non-small-cell lung cancer (NSCLC). Some studies [35] found that overexpression of miR-138 could inactivate lung cancer cell autophagy probably through the AMPK-mTOR signaling pathway, thus suppressing cell proliferation, invasion, and migration. In paclitaxel-resistant triple-negative breast cancer (TNBC) cells, miR-18a up-regulation enhanced autophagy via inhibiting the mTOR signaling pathway [36].

Paradoxically, either up-regulation or down-regulation of miR-96 suppressed PCa cell proliferation *in vitro* and tumor growth *in vivo* under hypoxia. Hypoxia increased the expression of miR-96 in PCa cells, and miR-96 stimulated autophagy by suppressing mTOR. But when ectopic overexpression of miR-96 reached a certain threshold, it abolished the hypoxia-induced autophagy through suppressing ATG7, a key autophagy-associated gene [37]. This observation might reveal a novel regulatory mode of autophagy by miRNAs. However, further studies are required to understand the complex role of miRNAs in autophagy regulation.

### Some miRNAs regulate drug-resistance related autophagy

Yu et al. [38] found that activation of the CXCL12/CXCR4 axis promoted epithelial–mesenchymal transition (EMT) and concurrent up-regulation of miR-125b in human CRC. Further experiments indicated a role of miR-125b in conferring 5-fluorouracil (5-FU) resistance in CRC, probably through increasing autophagy by augmenting the cleavage of L3-I into LC3-II both *in vitro* and *in vivo* [38]. *Beclin1*, a key autophagy-promoting gene, could be inhibited by miR-30d, which sensitized anaplastic thyroid carcinoma (ATC) cells to cisplatin [39] and promoted cell apoptosis of human colon cancer cells [40]. Colon cancer cells often become resistant during chemotherapy. Some recent studies [41] provided evidence that miR-409-3p targetted and inhibited Beclin1, which further inhibited chemotherapy-induced autophagy and enhanced the chemosensitivity of colon cancer cells.

In terms of tumor therapy, *low frequency magnetic fields* (*LF-MFs*), which refer to magnetic fields with 3–30 Hz, have been shown to inhibit cancer cell proliferation in several studies [42]. LF-MFs could up-regulate the expression level of miR-486 by targetting B-cell adaptor for phosphatidylinositol 3-kinase (BCAP) and further inhibiting lung cancer through miR-486-induced autophagic cell death by inhibiting AKT/mTOR signaling pathway [43]. Activation of the PI3K-Akt-mTOR pathway may trigger endocrine resistance to tamoxifen and fluvestrant in breast cancer, while miR-214 could increase the sensitivity of breast cancer cells to 4-OHT/FUL through inhibition of autophagy via the PI3K-Akt-mTOR pathway [44]. In addition, miR-142-3p could inhibit starvation-induced autophagy and increase chemosensitivity of NSCLC *in vitro* and *in vivo* through the PI3K-Akt-mTOR pathway [45]. An HIF-1α-miR-21 positive feedback loop was observed through the PTEN/Akt/HIF-1α pathway whereby miR-21 decreased autophagy, resulting in increased radio-resistance in cervical cancer cells [46]. Similarly, miR-140-5p expression was highly induced during chemotherapy of osteosarcoma cells, and this was accompanied by autophagy up-regulation. Importantly, miR-140-5p regulated this context-specific autophagy through its target *inositol 1,4,5-trisphosphate kinase 2* (*IP3k2*) and inhibited the IP3K2-mediated autophagy [47].

In spite of that, there are still many questions about the regulatory roles of miRNAs on autophagy that require further investigation. The same miRNA may have various influences on the different cancers through the dual function of autophagy, indicating that the exact role remains uncertain. Additionally, not all miRNAs concentrate on the levels of gene product, many of them only confirming to affect the entire network or signaling pathways. Therefore, future research should focus on a comprehensive understanding of the overall role of an miRNA in autophagy, thereby clarifying its role in tumorigenesis and development.

## Roles of lncRNAs in regulating autophagy

LncRNAs execute their functions by interactions with other components such as proteins, RNAs, and DNAs. RNA–protein, RNA–RNA, and RNA–DNA interactions could be combined by a single lncRNA to build distinct functionality complexes [48]. Guide-, decoy-, and scaffold functions of lncRNAs have been identified. The guide function of lncRNAs mediates recruitment of chromatin-modifying enzymes to target genes, thereby affecting gene transcription and expression. The decoy function involves binding of miRNAs through lncRNAs, combining with transcription factors to interfere with the binding of gene promoter region. As a scaffold or bridge for protein interaction, lncRNAs affected protein polymer formation and regulated protein activity [49]. In addition to their well-established influence as regulators of transcription, lncRNAs were also effective modulators of pre-mRNA splicing, mRNA decay, and translation [50].

### lncRNAs target autophagy-related proteins to regulate the autophagy

It is widely believed that *HOX transcript antisense RNA* (*HOTAIR*) mediates chromosomal remodeling and co-ordinates with polycomb repressive complex 2 (PRC2) to regulate gene expression in a variety of biological processes [51]. In hepatocellular carcinoma (HCC), HOTAIR was overexpressed in HCC tissues as compared with adjacent non-tumor tissues. HOTAIR overexpression could activate autophagy by increasing ATG3 and ATG7 expression, thus promoting HCC cell proliferation [52]. In addition, the expression level of HOTAIR was up-regulated in NSCLC. Further studies indicated that silencing of HOTAIR decreased drug resistance of NSCLC cells to crizotinib through inhibition of autophagy via suppressing phosphorylation of ULK1 which was an important component of ULK1–ATG13–FIP200 complex [53].

In papillary thyroid carcinoma (PTC), BRAF-activated lncRNA (BANCR) levels were significantly higher in those in PTC tissues, which contributed to PTC cell proliferation and activated autophagy [54]. This phenomenon was evaluated by observing the ratio of LC3-II/LC3-I, but we still donot know the specific regulation mechanism. In PTC, *GAS8 antisense RNA 1* (*GAS8-AS1*) was down-regulated, and overexpression of *GAS8-AS1* inhibited proliferation and activated autophagy by targetting ATG5 [55]. *LncRNA highly up-regulated in liver cancer* (*HULC*) could promote different pro-tumorigenic phenotypes, such as cell survival, proliferation, and invasion *in vitro*, as well as tumor growth and angiogenesis *in vivo* [56]. In epithelial ovarian carcinoma (EOC) tissues, the HULC expression level was higher than that in normal samples, which promoted tumorigenesis of ovarian carcinoma by inhibiting ATG7 to inhibit autophagy and induce progression by regulating ITGB1 [57]. HULC was also up-regulated in GC tissues and cell lines. However, up-regulation of HULC inhibited cell apoptosis by activating autophagy, and this overexpression was correlated with lymph node metastasis, distant metastasis, and advanced tumor node metastasis [58]. But, how HULC regulates autophagy and through which signaling pathway remains to be studied.

### LncRNAs target signaling pathways to regulate the autophagy

p53 is one of the most famous tumor suppressors and it has an outstanding role in promoting autophagic cell death. In PI3K-AKT signaling pathway, the activation of PI3K/AKT could inhibit p53 and inhibited autophagic cell death through mTORC1 activation [2]. In the cytoplasm, p53 exerted direct autophagy-inhibitory functions through a direct molecular interaction with the human ortholog of yeast ATG17, namely *RB1-inducible coiled-coil protein 1 (RB1CC1)*, also called FIP200, a protein that is essential for the very apical step of autophagy initiation [59]. Further investigations are required to better understand this dual aspect of p53 biology. *Maternally expressed gene 3* (*MEG3*) is an imprinted gene located at 14q32 that encodes an ncRNA [60], which is expressed in normal tissues but is either lost or decreased in many human tumors and tumor derived cell lines. Studies have demonstrated that MEG3 is associated with cancer initiation, progression, metastasis, and chemoresistance [61,62]. In bladder cancer tissues, MEG3 levels were significantly reduced, which inhibited cell apoptosis and increased cell proliferation by activating autophagy because MEG3 regulates cancer cell proliferation by the p53 pathway and p53 negatively regulates autophagy [63].

*Metastasis-associated lung adenocarcinoma transcript 1* (*MALAT1*) plays an important role in cancer and acts as a transcriptional regulator for various genes, including those involved in cell proliferation, migration, and metastasis [64]. In pancreatic ductal adenocarcinoma (PDAC), increased expression of MALAT1 was identified as a diagnostic biomarker. Silencing of MALAT1 inhibited autophagy via HuR-TIA-1-mediated autophagic activation, thus inhibiting tumor proliferation and metastasis *in vivo* [65]. TIA-1 functioned as ancient DNA/RNA transacting regulator to broaden the transcriptome and proteome diversity, which triggered a series of biological processes, including cell invasion, migration, apoptosis, and autophagy through regulating several p53 signaling pathway-related genes [66].

Although numerous studies have demonstrated the importance of PI3K in the activation of AKT, there have been reports suggesting that AKT activation can proceed in a manner that is independent of PI3K. Liu et al. [67] provided the evidence that LINC00470 was required for AKT cytoplasm activation and the interaction of LINC00470 and *fused in sarcoma (FUS)* was critical for AKT activation.High pAKT activated by LINC00470 inhibited cell autophagy, which associated with GBM tumorigenesis and poor patient prognosis [67]. In hypoxic tumor cells, lincRNA-p21 knockdown induced G_2_/M phase arrest, promoted apoptosis, decreased cell proliferation and motility, and reduced autophagy through HIF-1/Akt/mTOR/P70S6K pathway [68].

### Some lncRNAs regulate drug resistance related autophagy

In diffuse large B-cell lymphoma (DLBCL), MALAT1 was highly expressed. However, via elevating LC3-II/LC3-I protein expression and decreasing p62 expression, MALAT1 silencing could activate autophagy to inhibit adriamycin-induced EMT, thus suppressing tumor growth and reducing chemotherapy resistance [69]. In cisplatin-treated glioma cell line, MEG3 expression levels were increased. Elevated MEG3 enhanced the chemosensitivity of glioma cells to cisplatin through eliminating autophagy induced by cisplatin [70]. However, the molecular mechanisms underlying these effects remain unclear, and future studies will investigate this further.

## Co-ordinative roles of lncRNAs and miRNAs in regulating autophagy

LncRNA and miRNA can induce the occurrence of disease by multisite and multitarget interactions, and regulate these interactions by adjusting their relative abundance. Most importantly, lncRNA can act as a competing endogenous RNA (ceRNA) by combining the same miRNA with other RNA transcripts to degrade the target gene mRNA. This lncRNA–miRNA combination could regulate the gene expression pattern to realize the communication and regulation between them, and promote the physiological and disease processes, such as cell differentiation, proliferation, apoptosis, and the development and progression of diseases [71,72].

### LncRNA–miRNA axis targets autophagy-related proteins to regulate the autophagy

PVT1 was found to be highly expressed in glioma vascular endothelial cells. PVT1 overexpression increased the expression of ATG7 and Beclin1 by targetting miR-186, which induced protective autophagy, thus promoting glioma vascular endothelial cell proliferation, migration, and angiogenesis [73]. Additionally, MALAT1 expression was increased in glioma tissues compared with that in adjacent normal tissues. In addition, MALAT1 activated autophagy and promoted cell proliferation by sponging miR-101 and up-regulating STMN1, RAB5A and ATG4D expression in glioma cells [74].

HOTAIR expression was up-regulated in chondrosarcoma tissues and cell lines, which induced DNA methylation of miR-454-3p by recruiting EZH2 and DNMT1 to the miR-454-3p promoter regions to silence miR-454-3p expression. Through the HOTAIR-miR-454-3p-STAT3/ATG12 axis, HOTAIR knockdown-induced inhibition of autophagy indirectly promoted chondrosarcoma cell apoptosis [75]. *Small nucleolar RNA host gene 15* (*SNHG15*) contributed to invasion, proliferation, migration, and autophagy by negatively regulating miR-141 in osteosarcoma through elevating the levels of ATG5 and LC3-II. This finding may provide a new potential target and prognostic biomarker for the treatment of OS [76]. In HCC, HNF1A-AS1 functioned as an oncogene in tumor growth and apoptosis through sponging tumor-suppressive miR-30b-5p and de-repressing Bcl-2 and HNF1A-AS1-miR-30b axis significantly promoted autophagy under starvation [77]. ATG5, as a target of miR-30b-5p, was regulated by HNF1A-AS1-miR-30b axis, resulting in positively regulating the autophagy.

### LncRNA–miRNA axis targets signaling pathways to regulate the autophagy

In GC, lncRNA HAGLROS was a direct target of transcriptional factor STAT3 and could regulate autophagy-related gene expression of ATG9A and ATG9B by mTOR signals in two manners. On the one hand, HAGLROS acted as ceRNA of miR-100-5p to up-regulate mTOR expression by antagonizing miR-100-5p-mediated *mTOR* mRNA inhibition. On the other hand, HAGLROS could interact with mTORC1 components to activate mTORC1 signaling pathway. In these two manners, autophagy was inhibited, contributing to GC progression and poor prognosis [78]. LncRNA PTENP1 is a pseudogene of the tumor suppressor gene *PTEN* and its expression was down-regulated in several HCC cell lines. PTENP1 overexpression decoyed oncomirs miR-17, miR-19b, and miR-20a, which would otherwise target PTEN, suppress the oncogenic PI3K/AKT pathway, and induce autophagy and apoptosis [79].

### LncRNA–miRNA axis regulates drug resistance related autophagy

More recent evidence has indicated that protective autophagy is an important reason for chemoresistance of cancer cells [80]. Chemoresistance has long been recognized as a major obstacle in cancer therapy. In GC cells, MALAT1 acted as a ceRNA for miR-23b-3p and attenuated the inhibitory effect of miR-23b-3p on ATG12, leading to chemoinduced autophagy and chemoresistance in GC cells [81]. Treatment with antitumor reagents such as oxaliplatin, 5-FU, and pirarubicin (THP) dramatically induced HULC expression and protective autophagy in HCC cells. MiR-6825-5p, miR-6845-5p, and miR-6886-3p were down-regulated by HULC, resulting in the elevation of Sirt1, USP22, and protective autophagy, thus attenuating the sensitivity of HCC cells to chemotherapeutic agents [82]. In pancreatic cancer, blockade of autophagy could reduce pancreatic CSCs activity and sensitize cancer cells to gemcitabine [83]. In pancreatic cancer, linc-ROR confers gemcitabine resistance at least partly via inducing autophagy, and further research reported a linc-ROR/miR-124/PTBP1/PKM2 axis that involved in the regulation of gemcitabine resistance in pancreatic cancer cells [84].

*Myeloid cell leukemia sequence 1 (Mcl-1)*, an anti-apoptotic Bcl-2 family protein, has been reported to play a key role in autophagy. The anti-apoptotic Bcl-2 proteins including Bcl-2, Bcl-XL, Bcl-W, and Mcl-1 have been proposed to inhibit autophagy owing to their interaction with the autophagy regulator Beclin1 [85]. *LncRNA-AC023115.3* was increased in cisplatin-resistant glioma cells and acted as a ceRNA for miR-26a which attenuated the inhibitory effect of miR-26a on GSK3β, thus increasing GSK3β, a proline-directed serine-threonine kinase that promotes the degradation of Mcl-1, leading to an increase in GSK3β and a decrease in autophagy [86]. LncRNA, *cancer susceptibility candidate 2* (*CASC2*), was found as a tumor suppressor and down-regulated in various cancers [87]. *CASC2* was down-regulated in glioma, and overexpression of *CASC2* reduced TMZ-induced autophagy via mTOR up-regulation by targetting miR-193a-5p, thus enhancing the sensitivity of glioma cells to TMZ cytotoxicity [88]. Therefore, a better understanding about the molecular mechanisms of the interaction between lncRNA and miRNA would help enhance the protective autophagy and thus improve the outcome of chemotherapy for the treatment of cancers.

## Applications of autophagy in tumor therapy

Autophagy inhibition is receiving more and more attention as a potentially new therapeutic approach in cancer. This field is looking forward to the clinical development of novel agents that target the upstream components of autophagy or lysosomes. In the past few years, significant progress was made with the discovery of inhibitors of the two main kinases involved in the autophagy process, Vps34 and ULK1, and key regulator of the autophagy initiation, PI3K and mTOR. In addition, with the in-depth study of the regulation of autophagy by ncRNAs, making it possible to utilize of the ncRNA system in cancer biology and pathology by targetting autophagic pathways.

### Classical autophagy regulator

The seemingly paradoxical tumor-suppression and -promotion roles of autophagy provide more therapeutic opportunities for anticancer treatment. 3-methyladenine (3-MA), one of PI3K inhibitors, has been widely used as an autophagy inhibitor based on their inhibitory effect on class III PI3K activity [89]. But surprisingly, 3-MA was found to promote autophagy flux when treated under nutrient-rich conditions with a prolonged period of treatment due to its persistent inhibition on class I PI3K [90]. Understanding the dual role of 3-MA in autophagy may have important implications in autophagy study. In addition, hydroxychloroquine (HCQ) is the clinically available drug that could function as an autophagy inhibitor. HCQ inhibited autophagy by acting as a weak base and increased the pH of those compartments when trapped inside acidic cellular compartments (such as lysosomes) [91]. Rapamycin, an allosteric inhibitor of mTORC1, can selectively inhibit mTORC1 by binding to the 12-kDa immunophilin FK506-binding protein (FKBP12) to stimulate autophagy [92]. In addition to stimulating autophagic cancer cell death, the induction of autophagy by mTOR inhibitors could also sensitize cancer cells to other cancer therapeutics. But as we know, autophagy is important for some normal tissues, a critical question is whether systemic autophagy inactivation will be sufficiently selective to impair cancer growth while sparing normal tissues from the deleterious consequences.

### LncRNA and miRNA modulation in autophagy-targetted anticancer therapeutics

Recently, accumulating evidence has suggested that lncRNAs and miRNAs play critical roles in cell survival/death and therefore show great potential in regulating autophagy at different stages. As mentioned above, in different periods of autophagy, including vesicle nucleation, elongation, and completion phase, lncRNAs and miRNAs all promoted or inhibited the process of autophagy to a certain extent. Therefore, these results of the research enhanced the possibilities of the utilization of the lncRNA and miRNA system in cancer therapy by targetting autophagic pathways. For example, during the early stage of autophagy, miR-376b can decrease the activity of Beclin1, thereby blocking vesicle nucleation. In addition, miR-376b was able to attenuate mTOR inhibitor rapamycin-induced autophagy [93]. Thus, targetting miR-376b, we can develop relevant targetted therapies.

## Summary and future prospective

Autophagy is a classic mechanism of energy metabolism and self-renewal in cells, and plays an important role in biological development and homeostasis. Autophagy has a dual role in tumor therapy. As a tumor suppressor mechanism, autophagy can lead to cell death, limit the number of cells or reduce the mutation probability of DNA to prevent tumor formation. As a tumor protection mechanism, autophagy can protect cancer cells against chemotherapy drugs and delay apoptosis of tumor cells. However, there are still many problems to be solved in current research, such as the origin of autophagy, how signal transduction pathways co-ordinate and interact with each other, and their effects on cell survival.

It is well known that most cancer types can be cured, if diagnosed at an early stage. In recent years, many studies have shown that lncRNAs and miRNAs can be used as potential molecular diagnostic markers and therapeutic targets for disease. Their specific expression and regulation are closely related to many diseases, especially malignancies. The characteristics of lncRNA and miRNA, including disease specificity, cell type specificity and relative ease of detection, make them suitable for cancer patients. However, most ncRNAs are not tissue and cancer specific. For instance, HULC was dysregulated not only in EOC but also in GC. Furthermore, a group of patients with the same cancer type showed significant heterogeneity in ncRNA expression. Further studies should pay more attention to find more tissue and cancer-specific ncRNAs as potential molecular diagnostic markers and therapeutic targets. In addition, linking basic medical research with clinical treatment, namely translational medicine, is also the focus and difficulty in current scientific research. With the development of gene editing technology, especially the emergence of clustered regularly interspaced short palindromic repeats (CRISPR)/ RISPR associated (Cas), it is expected that more ncRNAs will be used as targets for clinical treatment.

**Figure 2:**
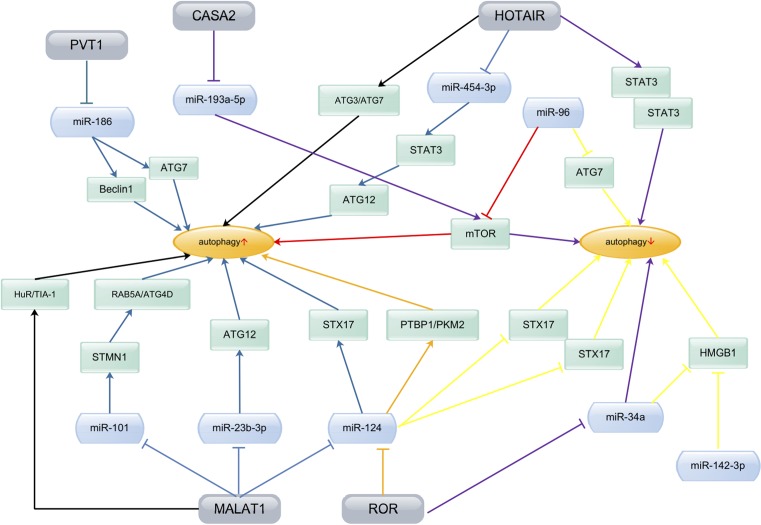
The complex network of lncRNAs and miRNAs in regulating autophagy Different ncRNAs regulate autophagy through diverse signaling pathway by targetting relevant proteins which ultimately result in different outcomes. ↑, increase; ↓, decrease.

In this review, many studies have demonstrated that lncRNAs and miRNAs play important roles in autophagy (Table 1), and the regulation of autophagy forms a complex network (Figure 2). At present, the research on the involvement of lncRNAs and miRNAs in autophagy is still in its initial stage. Although more autophagy-related lncRNAs and miRNAs have been discovered, most of these studies mainly focussed on their expression and function. Therefore, more studies are required to address the interaction mechanism between lncRNAs and miRNAs, and the regulatory complexity of autophagy, including specific target genes, target proteins, and signaling pathways, for the sake of helping discover new targets, new drugs or new strategies for the diagnosis, treatment, and prognostic prediction of cancers.

**Table 1:** A list of lncRNAs and miRNAs associated with autophagy regulation

LncRNA/miRNA	Expression level	Target	Promote/suppress autophagy	Cancer type	Reference
miR-18a	↑	mTOR	Promote	TNBC	[36]
miR-24-3p	↑	DEDD/p62	Promote	Bladder cancer	[20]
miR-152/miR-24	↑	Sirt1	Promote	Uterine sarcoma	[26]
miR-21	↑	Beclin1/Vps34/LC3II	Suppress	CML	[17]
miR-138	↓	AMPK/ mTOR	Suppress	Lung cancer	[35]
miR-30d	↓	ATG5/PI3K/Beclin1	Suppress	Colon cancer	[40]
HOTAIR	↑	ATG3/ATG7	Promote	HCC	[52]
HOTAIR	↑	ULK1	Promote	NSCLC	[53]
MEG3	↓	p53	Suppress	Bladder cancer	[63]
LINC00470	↑	AKT	Suppress	Glioblastoma	[67]

Abbreviations: ↑, up-regulation; ↓, down-regulation.

## Abbreviations

CASC2: *cancer susceptibility candidate 2*
ceRNA: competing endogenous RNA
CRC: colorectal cancer
CRISPR: clustered regularly interspaced short palindromic repeat
CSC: cancer stem cell
EMT: epithelial–mesenchymal transition
EOC: epithelial ovarian carcinoma
GAS8-AS1: *GAS8 antisense RNA 1*
GC: gastric cancer
HCC: hepatocellular carcinoma
HCQ: hydroxychloroquine
HOTAIR: *HOX transcript antisense RNA*
HULC: *highly up-regulated in liver cancer*
LC3: light chain 3
LF-MF: *low frequency magnetic field*
lncRNA: long non-coding RNA
MALAT1: *metastasis-associated lung adenocarcinoma transcript 1*
Mcl-1: *myeloid cell leukemia sequence 1*
MEG3: *maternally expressed gene 3*
MTMR3: *myotubularin related protein 3*
mTOR: mechanistic target of rapamycin
mTORC: mTOR complex
ncRNA: non-coding RNA
NSCLC: non-small-cell lung cancer
PCa: prostate cancer
PDAC: pancreatic ductal adenocarcinoma
PI3K: phosphoinositide 3-kinase
PTC: papillary thyroid carcinoma
Sirt1: Sirtuin 1
XIAP: *X-linked inhibitor of apoptosis*
3-MA: 3-methyladenine
5-FU: 5-fluorouracil
